# Human Papillomavirus Infection as a Possible Cause of Spontaneous Abortion and Spontaneous Preterm Delivery

**DOI:** 10.1155/2016/3086036

**Published:** 2016-03-27

**Authors:** Lea Maria Margareta Ambühl, Ulrik Baandrup, Karen Dybkær, Jan Blaakær, Niels Uldbjerg, Suzette Sørensen

**Affiliations:** ^1^Center for Clinical Research, North Denmark Regional Hospital and Department of Clinical Medicine, Aalborg University, Bispensgade 37, 9800 Hjørring, Denmark; ^2^Department of Hematology, Aalborg University Hospital, Søndre Skovvej 15, 9000 Aalborg, Denmark; ^3^Department of Obstetrics and Gynecology, Aarhus University Hospital, Palle Juul-Jensens Boulevard 99, 8200 Aarhus N, Denmark

## Abstract

Based on the current literature, we aimed to provide an overview on Human Papillomavirus prevalence in normal pregnancies and pregnancies with adverse outcome. We conducted a systematic literature search in PubMed and Embase. Data extracted from the articles and used for analysis included HPV prevalence, pregnancy outcome, geographical location, investigated tissue types, and HPV detection methods. The overall HPV prevalence in normal full-term pregnancies was found to be 17.5% (95% CI; 17.3–17.7) for cervix, 8.3% (95% CI; 7.6–9.1) for placental tissue, 5.7% (95% CI; 5.1–6.3) for amniotic fluid, and 10.9% (95% CI; 10.1–11.7) for umbilical cord blood. Summary estimates for HPV prevalence of spontaneous abortions and spontaneous preterm deliveries, in cervix (spontaneous abortions: 24.5%, and preterm deliveries: 47%, resp.) and placenta (spontaneous abortions: 24.9%, and preterm deliveries: 50%, resp.), were identified to be higher compared to normal full-term pregnancies (*P* < 0.05 and *P* < 0.0001). Great variation in HPV prevalence was observed between study populations of different geographical locations. This review demonstrates an association between spontaneous abortion, spontaneous preterm delivery, and the presence of HPV in both the cervix and the placenta. However, a reliable conclusion is difficult to draw due to the limited number of studies conducted on material from pregnancies with adverse outcome and the risk of residual confounding.

## 1. Introduction

Intrauterine infection by bacteria is well established as a pathway leading to spontaneous abortion and spontaneous preterm birth [1, 2]. Other pathways, however, may be equally important including decidual hemorrhage, cervical disorders, genetic components, and environmental exposures like smoking [3]. Much less is known about viral infection and adverse pregnancy outcome. Human Papillomavirus (HPV), which is known as a well-established cause for cervical cancer, does though constitute a candidate. The over 180 known HPV-types are small, double-stranded DNA viruses with a circular genome of nearly 8,000 base pairs. HPV infections are common, but about 90% of all infections can be cleared within less than 2 years by unknown mechanisms [4–6]. HPV-6 and HPV-11 are the most common low-risk types and are found to be causative for genital warts [6]. The cancer associated high-risk types include HPV-16 and HPV-18 [6] and there is growing evidence of HPV infections playing a relevant role in other anogenital and head and neck cancers [7–9]. Worth to mention is also the morbidity of cutaneous HPV lesions, particularly in immunosuppressed people [9].

Pregnancy has previously proven to be a state of mild immunosuppression due to the decrease in the number of natural killer cells [10], possibly making pregnant women more prone to infections with, for example, HPV. Various immunological theories have been discussed to explain the possibility for pregnancy and the survival of the “semiallogeneic” fetus. Theories include immunological privilege in the uterus, antigenic immaturity of the fetus, and maternal immunosuppression during pregnancy [11]. While attempting to explain the immunological basis of normal pregnancy, the argumentation may have implications for the generation of immune responses to pathogens infecting the placenta, as viruses seem to face similar confrontations like the invading trophoblast [11]. Thus, it is not surprising that viruses take up some of the same strategies to avoid immune detection as do trophoblast cells [12]. Also there is some evidence that elevated steroid hormone levels during pregnancy influence the increase of HPV virus replication by interacting with hormone response elements in the viral genome, thereby giving another possible explanation for the higher incidence of HPV infection during pregnancy [13, 14]. In 2014, Liu et al. [15] conducted a systematic review on HPV prevalence in pregnant and nonpregnant women and reported an increased risk of HPV infection in pregnant women, thereby supporting the debate of how far HPV may be involved in adverse pregnancy outcomes. Various authors report an infection with HPV during pregnancy to be associated with the risk of spontaneous abortion, spontaneous preterm delivery, and placental abnormalities [16–21]. HPV DNA has been detected in the cervix [13, 21–23], fetal membranes [24], amniotic fluid [25], umbilical cord blood [26, 27], and the placenta [13, 18, 27–29]. HPV detection rates range however widely from 6 to 65% and the results are controversial [13, 18, 20, 22, 23, 27, 30–32].

It is therefore of great interest to examine how widespread HPV infections are among pregnant women and whether or not there is an association between HPV infection and spontaneous abortion or spontaneous preterm delivery. Moreover, nowadays there exists a successful vaccination to prevent infection and disease caused by infection with HPV-6, HPV-11, HPV-16, and HPV-18. Thus, there might be a chance to minimize the risk for pregnancy complications by applying the same or a modified version of vaccination. Our group studies the impact of placental HPV infection on spontaneous abortion and preterm delivery. In this context the aim of this study is to provide an overview of the existing literature by doing a systematic review on HPV prevalence in pregnancy. We focused on pregnancies with adverse outcome and included a discussion of possible factors influencing or explaining the reported differences in HPV detection rates.

## 2. Material and Methods

PRISMA and MOOSE guidelines were used where applicable [33].

### 2.1. Search Strategy

A systematic literature search was conducted in the PubMed and Embase databases and search terms were used as follows: (1) “Human papillomavirus AND pregnancy”; (2) “Human papillomavirus AND preterm delivery”; (3) “Human papillomavirus AND preterm birth”; (4) “Human papillomavirus AND abortion”. The search was restricted to articles in English, on humans and published between January 1995 and October 2014. The search was carried out on October 28, 2014.

### 2.2. Study Selection

In order to identify relevant articles for whole-paper revision, duplicates were removed and titles and abstracts were screened based on the following exclusion criteria: studies investigating cell lines only, HPV vaccines, or sperm-related aspects, as well as guideline articles, general articles describing public health, and literature reviews. The remaining articles were assigned to subsequent whole-paper revision. These articles were systematically reviewed in accordance with the inclusion and exclusion criteria. The inclusion criteria were set to the following: studies on asymptomatic healthy pregnant women and women experiencing a spontaneous preterm delivery or spontaneous abortion; investigation of HPV infection within cervix, placenta, amniotic fluid, or umbilical cord blood; HPV detection test directly linkable to index pregnancy. Studies restricted to nonpregnant women or HPV positive women only, case reports, follow-up and association studies, and* in vitro* fertilization studies were excluded.

Studies including women with history of HPV-related lesions were not excluded, as this will be the case in any normal study population. There was no restriction for studies of different geographical origins, the time point of sample collection, methods of sample collection, and HPV testing method. Information on the latter was included in Table 1, characteristics of included studies.

### 2.3. Data Extraction and Statistics

Data, which includes information about the investigated tissue types, country where the study was performed, sample size, sampling time point, HPV prevalence, and HPV detection method, was extracted and analyzed using MatLab (version R2011b). Data from all studies was pooled and women were grouped according to their pregnancy outcome or the pregnancy status at time point of sample collection. An overall HPV prevalence was calculated including 95% confidence interval (CI) in normal pregnancies and in pregnancies with adverse outcome. This was done for various tissue types, geographical origins, time points of sample collection, and HPV detection methods used. Studies from Mexico and Brazil have been grouped into “Latin America.” Statistical significance between two proportions was tested using “Two-sample test of proportions.” All *P* values were two-sided and *P* < 0.05 was considered significant.

## 3. Results

### 3.1. Study Selection

**Figure 1:**
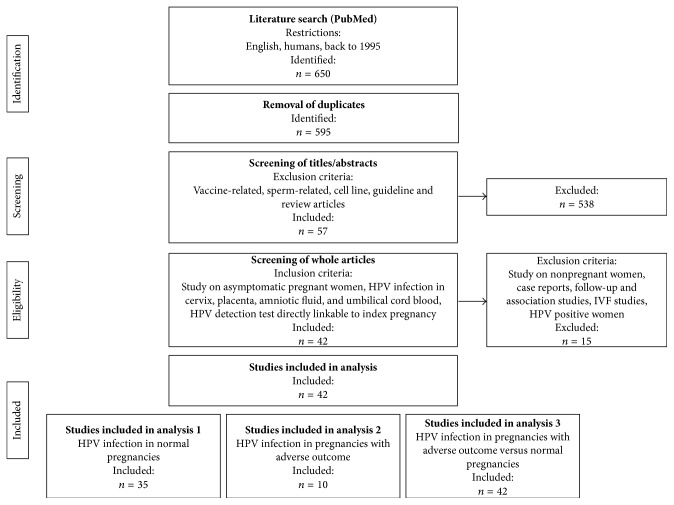
Flow diagram of literature search. The flow diagram shows the search in the PubMed database. A supplementary search in the Embase database was conducted in the same way and resulted in three additional articles for data extraction and quantitative analysis.

The initial PubMed database search resulted in the identification of a total of 650 articles (Figure 1). After removal of duplicates and screening of titles and abstracts, the remaining 57 articles were subjected to whole-paper revision (Figure 1). Of these, 42 articles met the final inclusion criteria and were used for data extraction and quantitative analyses (Figure 1). The supplementary search in Embase database was conducted in the same way and three additional articles were included for data extraction and quantitative analysis. The 45 articles included investigated 14 470 pregnant women in total, of which 13 757 underwent normal full-term pregnancies, 145 experienced spontaneous preterm deliveries, 536 experienced spontaneous abortions, and 32 had performed an induced abortion. The study populations were from Europe (*n* = 4639) [13, 22, 27, 28, 32, 38, 40, 42, 43, 45, 47, 51, 58–60, 62–64, 66, 67], Asia (*n* = 7116) [23, 34, 35, 37, 39, 41, 46, 52, 55, 57, 61], USA (*n* = 1681) [18, 20, 21, 29, 48, 53, 56, 65, 68], and Latin America (*n* = 1034) [36, 44, 49, 50, 54]. Table 1 shows demographic characteristics, key aspects of study design, and study strength and potential biases of all included studies.

### 3.2. HPV Prevalence in Different Tissue Types of Normal Full-Term Pregnancies

**Figure 2:**
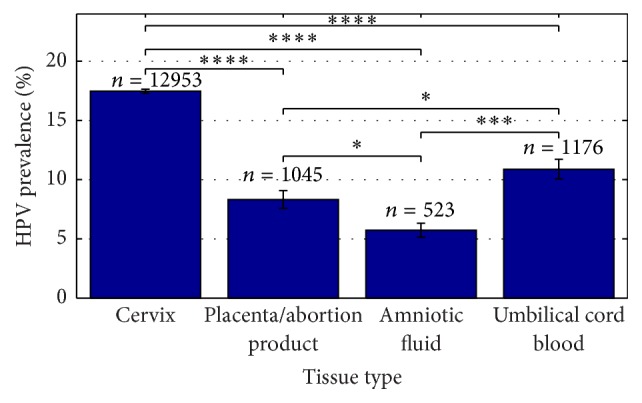
HPV prevalence depends on the investigated tissue type. HPV prevalence in different tissue types of normal pregnancies in %. 38 studies have been included in the present analysis, *N*_cervix_ = 32, *N*_placenta/abortion  product_ = 9, *N*_amniotic  fluid_ = 4, and *N*_umbilical  cord  blood_ = 7. *N* indicates number of studies included. *n* indicates number of cases included. ^*∗*^ *P* < 0.05, ^*∗∗∗*^ *P* < 0.001, and ^*∗∗∗∗*^ *P* < 0.0001.

HPV prevalence in healthy pregnant women giving birth at term was investigated in 38 of the 45 included studies and provided data on 13 757 pregnant women. HPV prevalence appears to be highly dependent on the tissue type tested (Figure 2). In all studies included, HPV prevalence varied between 2.2% and 75% in cervical tissue, with a summary estimate of 17.5% (95% CI; 17.3–17.7). In placental tissue and abortion products, 8.3% (95% CI; 7.6–9.1) of the analyzed pregnancies were found to be HPV positive and varied between 0% and 47.2%. HPV prevalence in amniotic fluid varied between 0% and 25%, with a summary estimate of 5.7% (95% CI; 5.1–6.3). Finally, umbilical cord blood was calculated to be HPV positive in 10.9% (95% CI; 10.1–11.7) of all cases and varied between 0% and 57.9%. The difference between all proportions was significant (*P* < 0.05, *P* < 0.001, and *P* < 0.0001, resp.).

### 3.3. HPV Prevalence in Pregnancies with Adverse Outcome and Comparison to Normal Pregnancies

HPV prevalence in pregnancies with adverse outcome, including spontaneous abortion and spontaneous preterm delivery, was investigated in 10 of the 45 included studies and provided data on 681 pregnancies. Three studies were investigating cervical HPV infection and seven studies looking at HPV prevalence in placental tissue. Details are given in Table 2. Only one study analyzed HPV prevalence in cervical tissue of spontaneous abortions and found 24.5% of all cervical samples to be HPV positive (Table 2). Placental HPV prevalence in spontaneous abortions varied between 0% and 70.4%, with a summary estimate of 24.9% (95% CI; 22.4–27.5) (Table 2). HPV prevalence in spontaneous preterm deliveries was found to be 47% (95% CI; 42.3–51.6) in cervix, with a variation between 15.6% and 67.1% (Table 2). Placental tissue of spontaneous preterm deliveries was only investigated in one study where a HPV prevalence of 50% was observed (Table 2).

**Figure 3:**
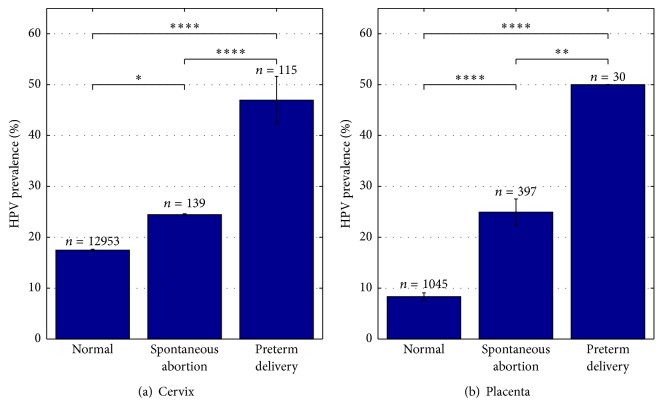
Higher HPV prevalence detected in pregnancies with adverse outcome compared to normal pregnancies. HPV prevalence in normal pregnancies, spontaneous abortions, and spontaneous preterm deliveries in %. (a) In cervix. 34 studies have been included in the present analysis, *N*_Normal_ = 32, *N*_Spontaneous  abortion_ = 1, and *N*_Preterm  delivery_ = 2. (b) In placenta. 14 studies have been included in the present analysis, *N*_Normal_ = 9, *N*_Spontaneous  abortion_ = 6, and *N*_Preterm  delivery_ = 1. *N* indicated number of studies included. *n* indicates number of cases included. ^*∗*^ *P* < 0.05, ^*∗∗*^ *P* < 0.01, and ^*∗∗∗∗*^ *P* < 0.0001.

The overall HPV prevalence in cervical tissue of normal pregnancies was found to be 17.5% (95% CI; 17.3–17.7) (Figure 2). This is significantly lower than in spontaneous preterm deliveries (47%, *P* < 0.0001) and spontaneous abortions (24.5%, *P* < 0.05) (Figure 3). An even bigger contrasting picture is seen in placental tissue. Here normal pregnancies are found to be HPV positive in 8.3% (95% CI; 7.6–9.1) of all cases (Figure 2), whereas placental tissues of spontaneous preterm deliveries and spontaneous abortions are found to be positive in 50% (*P* < 0.0001) and 24.9% (*P* < 0.0001) of cases, respectively (Figure 3).

### 3.4. HPV Prevalence in Normal Pregnancies Depends on Geographical Location

Information on geographical distribution of the investigated studies was collected to see if ethnicity might be a contributing factor to the great variation in HPV prevalence observed. The goal of the investigation was to determine whether women from different countries may be differentially exposed to HPV and some may thereby have a higher risk for possible HPV-induced pregnancy deficiencies.

**Figure 4:**
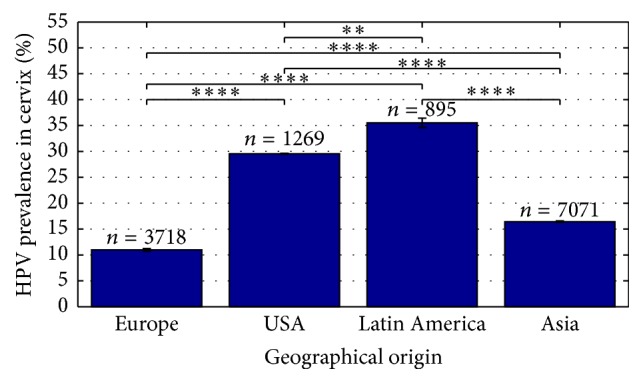
HPV prevalence in normal pregnancies depends on geographical location. HPV prevalence in cervix of normal pregnancies in %. 32 studies have been included in the present analysis, *N*_Europe_ = 13, *N*_USA_ = 4, *N*_Latin  America_ = 5, and *N*_Asia_ = 10. *N* indicated number of studies included. *n* indicates number of cases included. ^*∗∗*^ *P* < 0.01, ^*∗∗∗∗*^ *P* < 0.0001.

Figure 4 shows the geographical distribution of HPV prevalence in cervical specimens from women with normal full-term pregnancies. Here it is clear that pregnant women from USA and Latin America have a significantly higher (*P* < 0.0001) HPV prevalence compared to European and Asian women (Figure 4). Latin America is represented by Mexico and Brazil and the summary estimate for HPV prevalence was 35.5% (95% CI; 34.6–36.5). HPV prevalence found in the included studies varied between 15.2% and 75%. The analysis of the population from USA found a HPV prevalence of 29.6% (95% CI; 29.5–29.7). Here a variation of 28% to 34.2% has been reported. The Asian population is represented by China, Japan, and Korea. The cervical HPV prevalence in Asia varied between 10.1% and 36.2%, with a summary estimate of 16.4% (95% CI; 16.3–16.6). The European population is represented by Spain, France, Italy, Germany, Belgium, Netherlands, Finland, Switzerland, Austria, Hungary, Greece, Croatia, Turkey, Poland, and Lithuania and the HPV prevalence varied between 2.2% and 36.6%, with a summary estimate of 11% (95% CI; 10.7–11.3). The difference between all proportions was highly significant (*P* < 0.01 and *P* < 0.0001, resp.), meaning that HPV prevalence in pregnant women is dependent on geographical or ethnical parameters, with pregnant women from USA and Latin America having the highest HPV prevalence reported. The same tendency can be observed in placenta, amniotic fluid, and umbilical cord blood, but numbers were insufficient for proper analyses.

Variation of HPV prevalence between studies conducted on different continents can also be observed in studies investigating pregnancies with adverse outcome (Table 2), but a proper analysis is difficult due to the small number of studies. However, it can be stated that studies from USA consistently report a significantly higher HPV prevalence in spontaneous abortions and spontaneous preterm deliveries compared to normal pregnancies, in both cervical and placental tissue [18, 20, 21, 29]. European studies vary a lot in their HPV prevalence found in placenta, which makes it difficult to estimate the risk of a HPV infection for European pregnant women. There are no studies conducted on cervical specimens of spontaneous abortions or spontaneous preterm deliveries in Europe. Asian and Latin American studies are also limited [35, 36] and a conclusion of the risk of HPV infection for pregnant women is not possible. It can though be speculated whether the relatively high HPV prevalence found in the Latin American population of normal pregnancies (Figure 4) may influence the pregnancy outcome in those countries.

### 3.5. Influence of the Time Point of Sample Collection and the HPV Detection Method on HPV Prevalence

**Figure 5:**
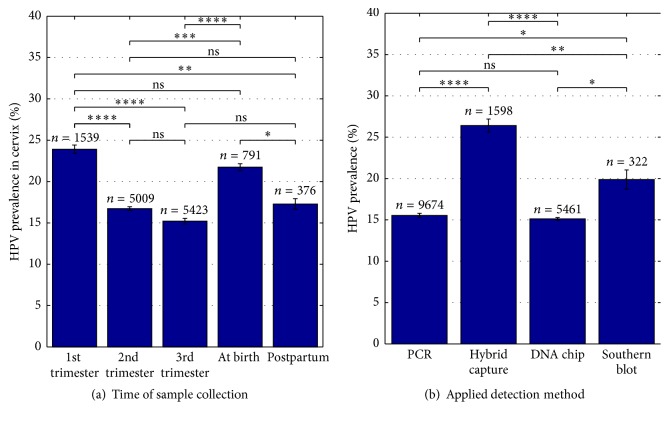
HPV prevalence depends on time of sample collection and on the applied HPV detection method. (a) Cervical HPV prevalence in % at different time points of sample collection of normal pregnancies. 25 studies have been included in the present analysis, *N*_1st  trimester_ = 10, *N*_2nd  trimester_ = 10, *N*_3rd  trimester_ = 18, *N*_at  birth_ = 4, and *N*_postpartum_ = 4. (b) HPV prevalence in % in relation to the detection method used. Note that only two studies were using Southern blot as their main detection method. 45 studies have been included in the present analysis, *N*_PCR_ = 34, *N*_hybrid  capture_ = 8, *N*_DNA  chip_ = 5, and *N*_Southern  blot_ = 2. *N* indicated number of studies included. *n* indicates number of cases included. ^*∗*^ *P* < 0.05, ^*∗∗*^ *P* < 0.01, ^*∗∗∗*^ *P* < 0.001, and ^*∗∗∗∗*^ *P* < 0.0001; ns: not significant.

There are multiple factors that may influence HPV prevalence. For the present analysis, data on the time point of sample collection and the HPV detection methods used were collected.  Figure 5(a) shows cervical HPV prevalence in relation to the time point of sample collection. Samples from the first trimester of pregnancy were found to be HPV positive in 23.9% (95% CI; 23.4–24.4) of all cases and showed a variation between 1.1% and 41.2%. Samples taken at birth were tested positive in 21.7% (95% CI; 21.3–22.2) of all cases. Here HPV prevalence varied between 12.6% and 30.2%. The second and third trimester as well as postpartum samples showed a HPV prevalence of 16.7% (95% CI; 16.5–17.0), 15.2% (95% CI; 14.9–15.6), and 17.3% (95% CI; 16.7–17.9). HPV prevalence varied between 2.2% and 40%, 5.2% and 75%, and 6.2% and 27%. The difference between proportions was significant (*P* < 0.05, *P* < 0.01, *P* < 0.001, and *P* < 0.0001 resp.). Not significant was the difference between the proportions for the first trimester and at birth samples (*P* = 0.12), the second trimester and the third trimester, respectively, and the postpartum period (*P* = 0.07 and *P* = 0.39), and the third trimester and the postpartum period (*P* = 0.14).

Also the HPV detection methods used may influence the found HPV prevalence.  Figure 5(b) shows the analysis of HPV prevalence according to the HPV detection method used. Hybrid capture assay identified HPV in 26.4% (95% CI; 25.6–27.2) of all analyzed samples. The HPV prevalence found varied between 0% and 67.1%. The summary estimate for HPV prevalence by PCR was calculated to be 15.5% (95% CI; 15.3–15.8) and varied between studies from 0% to 100%. HPV prevalence identified by DNA chip varied between 0% and 24.3%, with a summary estimate of 15.1% (95% CI; 15.0–15.3). Southern blotting was only used in two studies and a HPV prevalence of 19.9% (95% CI; 18.7–21.0) has been found. HPV positivity varied between studies from 12% to 34.2%. The difference between all but one of the proportions was significant (*P* < 0.05, *P* < 0.01, and *P* < 0.0001, resp.). Not significant was the difference between proportions for PCR and DNA chip (*P* = 0.25). The HPV detection method used most widely was PCR with *n* = 9674 followed by DNA chip with *n* = 5461.

## 4. Discussion

In this quantitative analysis on the prevalence of HPV infection in normal pregnancies and pregnancies with adverse outcome, 45 studies were included and data on 14 470 pregnant women were analyzed and summarized. HPV prevalence in normal pregnancies was found to vary between tissue types and study populations of different geographical locations. The highest HPV prevalence could be reported in cervix (17.5%; 95% CI; 17.3–17.7) and in the population from Latin America (35.5% (95% CI; 34.6–36.5)) and USA (29.6% (95% CI; 29.5–29.7)). In comparison to HPV prevalence found in normal pregnancies, spontaneous abortions and spontaneous preterm deliveries were found to have higher HPV positive detection rates (*P* < 0.05 and *P* < 0.0001), in both placenta (spontaneous abortions: 24.9%, and preterm deliveries: 50% versus 8.3%, resp.) and cervix (spontaneous abortions: 24.5%, and preterm deliveries: 47% versus 17.5%, resp.). Beyond the geographical location, the time point of sample collection in pregnancy as well as the HPV detection methods used may influence the results on HPV prevalence.

The present work has some weaknesses. First, heterogeneity between studies is a problem. Due to the limited number of studies conducted within the field of HPV infections and adverse pregnancy outcomes, inclusion criteria were set relatively widely. This results in a higher number of pregnancies to analyze but possibly more heterogeneous study groups and thereby inflict restricted options to compare directly between studies. The possibility of forming totally homogenous study groups is limited by the study quality and information given, and data on patient-level is missing in most, if not all, of the included studies, which makes controlling for all potential biases difficult. Table 1 contains potential biases and study strength for every single study. Potential biases may include some of the following: unclear inclusion and exclusion criteria for participating women, missing information on ethnicity/method of sample collection/time point of sample collection/mode of delivery, study on highly selected group of women (cesarean sections, amniocentesis, and CVS) or group from low socioeconomically regions, and so forth. The interconnection between HPV infection and pregnancy outcome is complex and will therefore most probably not be explainable by a single parameter analyzed in the present work. The analyses done can however point towards a possible explanation by pregnancy outcome, geographical location, or HPV detection method. Second, studies vary in their inclusion of women with histories of HPV-related diseases. Approximately one-third excludes women with HPV-related lesions, whereas one-third does not exclude but report it and in the last third of studies this information is missing. Genital warts and cervical lesions are known to be HPV-related and a higher percentage of HPV infection would be expected. Third, studies of adverse pregnancies were few. The actual HPV prevalence may therefore be higher or lower than the ones reported in this paper. On the other hand, the present quantitative analysis includes over 14 000 pregnancies and covers many different factors possibly influencing HPV prevalence, thereby providing a broad overview. Fourth, only studies published in English were included, which might limit the results. However, English is the primary common scientific language and the selection criterion “publications in English” is therefore considered to be acceptable.

HPV prevalence in pregnant women has been reported to be higher compared to nonpregnant women [54, 68, 69]. Our analysis of normal pregnancies, including 38 studies from 19 different countries on four continents, yields an overall HPV prevalence in the cervix for pregnant women of 17.5% (95% CI: 17.3–17.7). A worldwide meta-analysis by de Sanjosé et al. from 2007 reported a prevalence of cervical HPV infections in nonpregnant women with normal cytology of 10.4% [70]. The difference between the proportions is noticeable and it can therefore be speculated if a higher HPV prevalence in pregnant women can lead to various pregnancy complications. Pregnancy has previously been described as a state of immune suppression, facilitating the survival of the “semiallogeneic” fetus [52]. The inhibition of the host immune response may simultaneously increase the susceptibility to HPV and make it more difficult to clear HPV infections [54, 69, 71–74]. Higher incidences of HPV infection during pregnancy may though possibly be explained by the presence of hormone response elements in the HPV gene that could be triggered through high steroid levels [13, 14].

An infection with HPV during pregnancy has been associated with risk for spontaneous abortion and spontaneous preterm delivery as well as placental abnormalities [16–21]. Our analysis of 10 studies investigating HPV infection in pregnancies with adverse outcome found HPV prevalence in cervix and placenta to be higher than in normal full-term pregnancies, supporting the hypothesis of HPV infection being a risk for pregnancy outcome. Typically the placenta is a relatively effective barrier guarding against many microbes [75, 76] and the fetus* in utero* is therefore well protected. Nevertheless, multiple studies indicate that viral infection can impair trophoblast function, potentially contributing to pregnancy loss or abnormal implantation [76, 77]. Furthermore, upper genital tract infection was found to be an important cause of preterm birth [78].

As previously mentioned, the wide range of HPV prevalence found is related to several factors including study design, geographic and demographic characteristics, choice of detection method, and risk factor profiles, such as maternal age, gestational age, and history of cesarean section [31, 35, 70, 79]. The present work underlines the importance to consider geographical and demographic variances. The HPV prevalence around the world was found to differ significantly (11–35.5%) with Latin America having the highest cervical HPV prevalence in pregnant women. This is well in line with the previous literature where Latin America and Africa are reported as the geographical areas with the highest cervix cancer rate in the world [8]. North American studies do in general report a relatively high HPV prevalence [20, 48, 56, 65, 68] and our analysis found a HPV prevalence of 29.6% (95% CI; 29.5–29.7) for USA. This may be a bit puzzling since the population in North America due to historical reasons is expected to resemble the population in Europe. It can therefore be speculated that studies conducted in North America include a distinct number of the Latin American and African American population as has been stated by Gomez et al. in 2008 [20]. Unfortunately information of ethnicity of participating women is not available for many of the included studies, providing a potential bias to the results presented on geographical origin.

The time point of sample collection during pregnancy is thought to have an influence on HPV prevalence as differences in HPV prevalence regarding the trimester tested have been published [80–83]. Our analysis involved over 13 000 samples and summarized many of the studies investigating the topic. Hereby the first trimester was found to have the highest overall HPV prevalence, closely followed by the samples taken at birth. Analyzed samples collected in the second and third trimester as well as in the postpartum period showed lower HPV prevalence. This is contradictory to studies reporting highest HPV prevalence during the second [34] and third [54, 65, 68] trimester. On the other hand, multiple HPV infections were observed in the first trimester by Yamasaki et al. in 2011 and high-risk HPV-types were found to be selectively increased in the first trimester as well [46, 68]. It is however important to keep in mind that all those time points in reality are time intervals, thereby making a comparison very difficult. To reduce potential bias due to uncertainty about the time point of sample collection, we have chosen to include only studies providing information on the time point of sample collection. This is the case for 25 of the 45 included studies.

Finally, we compared HPV prevalence with respect to choice of HPV detection methods, since studies have shown that HPV DNA detectable and the HPV-genotypes identified largely depend on this [42]. Our analysis of HPV prevalence in regard to the applied detection methods revealed hybrid capture assay as the method detecting most HPV positive cases (Figure 5(b)). PCR is thought to be the most sensitive method, detecting less than 10 copies of HPV DNA [46, 84, 85]. In our analysis the HPV prevalence detected by PCR was found to be 15.5% (95% CI; 15.3–15.8). Currently an increasing number of studies use broad spectrum primers [26, 31, 56] instead of type-specific primers. These are designed to detect various HPV-types at the same time, hereby giving a broader picture of the HPV-types present. The downside of high sensitivities allowed by PCR is the risk of false-positives due to contamination [84]. It is therefore crucial to evaluate tissue samples on different molecular levels, confirming the detected virus to be correctly localized and active, thus being able to explain the causality of adverse pregnancy outcome or diseases in general.

Functional studies investigating HPV infection in relation to pregnancy are limited. Following the detection of HPV in placental tissue [17, 18, 62] most research is focused on the study of HPV infections in trophoblast cell lines. Being one of the most critical tissues of the placenta, the trophoblast layer plays an important role in contacting maternal tissues and serves multiple roles during gestation. Several lines of evidence point towards the trophoblasts as being the target cells of placental HPV infection. First, it was shown that HPV is able to undergo a complete life cycle in trophoblast cell lines [86, 87]. Second, trophoblast morphology and behavior during HPV infection have been investigated, reporting a higher rate of apoptosis and lower invasion capabilities, agreeing with possible placental dysfunction and adverse pregnancy outcome [20]. Third, studies analyzing trophoblast cells transfected with the HPV genes E5, E6, and E7 report effects on trophoblastic adhesion and increased migratory and invasive properties and may eventually explain potential abnormal implantation due to inappropriate trophoblast spreading [88]. However, a convincing role for HPV infection in connection with spontaneous abortion and spontaneous preterm delivery, at the molecular level, has still to be demonstrated.

## 5. Conclusion

Based on the present quantitative analyses it can be concluded that HPV prevalence is higher in pregnancies with adverse outcome, such as spontaneous abortion or spontaneous preterm delivery, compared to women experiencing a normal full-term pregnancy. HPV infection may therefore be constituted as a risk for the present pregnancy.

HPV prevalence has been shown to be dependent on the tissue type tested and the geographical location of the study population analyzed. However, the number of studies investigating HPV infection on material from spontaneous abortions and spontaneous preterm deliveries is very limited and study groups are heterogeneous which makes a reliable conclusion difficult. It can be stated that study design is important, the selection of proper controls is essential, and, for a valuable comparison between studies, similarity in samples/patients needs to be controlled as strictly as possible. Furthermore, one should keep in mind that the simple detection of a virus never is equal to a real causative role in the adverse outcome of a pregnancy or diseases in general. It is therefore inevitable to study the viral activity and cellular localization to be able to conclude on the impact for a given situation. Therefore we recommend including an investigation of the molecular mechanism of HPV infections in material of pregnancies with adverse outcome and inviting researchers to conduct new studies to clarify HPV's impact on spontaneous abortion and spontaneous preterm delivery. As a consequence of the aforementioned limitations our study group has initiated a prospective study addressing the complexity of HPV in pregnancy.

## Figures and Tables

**Table 1:** Characteristics of included studies.

References	Country	Inclusion criteria	Gestational age at sample collection (weeks)	Examined tissue type	Sample size	HPV prevalence%	HPV detection method	Study quality	
								Study strengths	Potential biases
[34]	Korea	Pregnant women healthy, ≥18 years of age, sonographically confirmed intrauterine pregnancy	1st, 2nd, or 3rd trimester or postpartum	Cervix	Total: 960 1st trimester: 380 2nd trimester: 193 3rd trimester: 195 Postpartum: 192	Total: 24.3 1st trimester: 20.5 2nd trimester: 34.2 3rd trimester: 23.1 Postpartum: 22.9	DNA chip	Large cohort, multivariant logistic regression analysis, separate analysis for trimesters	Sample collection at different time points, including women with abnormal pap smear

[35]	Korea	PD healthy, <37 weeks of gestation	6-week postpartum	Cervix	45	15.6	HCA	Multivariant logistic regression analysis	Small sample size, postpartum sampling only, HCA sensitivity limited to 13 HPV-types

[36]	Mexico	SA: healthy, curettage up to week 20 ND: healthy, attending for delivery at term with viable products	SA: <20 ND: before delivery	Cervix	SA: 139 ND: 138	SA: 24.4 ND: 15.2	PCR	Large cohort, risk analysis	Comparison of SA (first trimester) with ND (at term), HPV-related disease history unclear

[37]	Korea	Pregnant women healthy, singleton, in first trimester	Cervix: 1st, 2nd, and 3rd trimester, postpartum Placenta, umbilical cord blood: at birth	Cervix, placenta, umbilical cord blood	Cervix: 153 Placenta: 152	Cervix: 24 Placenta: 3.3 Umbilical cord blood: 1.3	DNA chip	Large cohort, longitudinal follow-up, multivariant logistic regression analysis	Sample collection from cervix at different time points

[38]	Poland	Pregnant women singleton, normal cervical smear	33–41	Cervix	135	16.3	PCR	Large cohort, multivariant logistic regression analysis, confirmation by sequencing	Vaginal and cesarean deliveries included, potential contamination problem (HPV11 present in all positive samples)

[23]	Korea	Pregnant women healthy, over 36 weeks of gestation	>36	Cervix, placenta, umbilical cord blood	469	Cervix: 15.4 Placenta: 0 Umbilical cord blood: 0	DNA chip	Large cohort, confirmation by *in situ* hybridization	Vaginal and cesarean deliveries included Umbilical cord blood and placenta were collected from HPV positive mothers

[39]	China	Pregnant women healthy, asymptomatic	22.5–26.7	Cervix	3139	13.4	DNA chip	Large cohort, logistic regression analysis	Vaginal and cesarean deliveries included, women with abnormal cervical cytology included

[40]	Netherlands	Pregnant women NA	1st, 2nd, and 3rd trimester, postpartum	Cervix	51	21.6	PCR	Matched groups, detection method with high analytical sensitivity	Small sample size, self-sampling, sample collection at different time points

[41]	Korea	Pregnant women healthy, over 36 weeks of gestation	>36	Cervix	291	18.9	DNA chip	Large cohort, stratified analysis to test for confounding	Women with abnormal cervical cytology included, vaginal and cesarean deliveries included

[42]	Finland	Pregnant women healthy, in third trimester	3rd trimester, at birth	Cervix, placenta, umbilical cord blood	Cervix: 329 Placenta: 306 Umbilical cord: 311	Cervix: 16.4 Placenta: 4.2 Umbilical cord blood: 3.5	PCR	Large cohort, regression analysis, multimetrix assay for HPV detection, pap smear at baseline	—

[43]	Poland	SA: healthy ND: healthy, delivering at term	SA: 6–16 ND: at birth	Aborted products of conception, placenta	SA: 51 ND: 78	SA: 17.7 ND: 24.4	PCR	—	Small sample size, comparison of SA with ND, HPV-related disease history unclear

[44]	Mexico	Pregnant women healthy, in third trimester, delivering at term	3rd trimester	Cervix, placenta	72	Cervix: 75 Placenta: 47.2	PCR	PCR process was blinded	Potential contamination problem (HPV18 present in all positive placenta samples), vaginal and cesarean deliveries included

[45]	Lithuania	Pregnant women NA	1st and 3rd trimester	Cervix	1st trimester: 213 3rd trimester: 146	1st trimester: 17.8 3rd trimester: 10.3	PCR	Large cohort, risk analysis, separate analysis for trimesters	Big proportion with history of gynecological diseases, exclusion of 67 women due to change of residency/miscarriage/ premature delivery, no inclusion and exclusion criteria, commercial HPV PCR kit

[46]	Japan	Pregnant women NA	1st, 2nd, or 3rd trimester or postpartum	Cervix	151	35.8	PCR	Large cohort, pap smear at study entry	Sample collection at different time points, unclear how and when women deliver

[21]	USA	PD <37 weeks of gestation, available HPV-test results	NA	Cervix	70	67.1	HCA	Risk analysis (age, race), data from over 11 years	Sampling method and time point not mentioned, study including African Americans, HCA restricted to 13 HPV-types only

[47]	Turkey	Pregnant women NA	18–22	Cervix	134	2.2	PCR	Large cohort	22 with abnormal ultrasound findings, other virus types being in focus, HPV-related disease history unclear, no inclusion and exclusion criteria, outpatient clinic (low socioeconomic group)

[32]	Belgium	Pregnant women assigned for abdominal CVS, mainly due to high risk for chromosomal abnormalities	11–13	Placenta	35	5.7	PCR	Transabdominal sampling (no birth canal contamination), highly sensitive detection method, confirmation by sequencing	Small sample size, highly selected group of women, limited amount of placenta material (actual HPV prevalence higher?), HPV-related disease history unclear

[48]	USA	Pregnant women healthy, in third trimester, 18 and above	3rd trimester	Cervix	333	28	PCR	Large cohort, logistic regression analysis, confirmation by sequencing	Vaginal and cesarean deliveries included, unclear if deliveries are at term, 25% of women with history of HPV-related lesions

[49]	Brazil	Pregnant women NA	2–37	Cervix	371	35.3	HCA	Large cohort, multivariant logistic regression analysis	Inclusion at ambulatories for patients suspected to infectious diseases, no inclusion and exclusion criteria, sample collection at different time points, women with genital warts included

[50]	Brazil	Pregnant women NA	NA	Cervix	40	25	PCR	—	Small sample size, inclusion at outpatient clinic (low socioeconomic group), no inclusion and exclusion criteria, HPV-related disease history unclear, sampling time point not mentioned

[51]	Spain	Pregnant women unselected	29–33	Cervix	828	6.5	PCR	Large cohort, multivariant logistic regression analysis	Goal to find HPV positive women for prospective cohort study on mother-to-child transmission, HPV-related disease history unclear, no inclusion and exclusion criteria, vaginal and cesarean deliveries

[27]	Finland	Pregnant women third trimester	31.6–42.5	Placenta, Umbilical cord blood	Placenta: 306 Umbilical cord blood: 311	Placenta: 4.2 Umbilical cord blood: 3.5	PCR	Large cohort, multivariant regression analysis, pap smear at study entry, confirmation by sequencing	Included women delivering before week 37, part of women showing genital warts or cervical lesions, no HPV status examination before recruitment

[20]	USA	PD: spontaneous, <37 weeks of gestation ND: delivering at term	PD: 21–36 ND: 37–42	Placenta	PD: 30 ND: 30	PD: 50 ND: 20	PCR	Comparison of PD to ND (best possible control), confirmation by sequencing	Small sample size, study including mostly African Americans, HPV-related disease history unclear, type-specific PCR only

[29]	USA	SA: singleton, in second trimester IA: singleton, for congenital anomalies or maternal medical indications	16.7–23.6	Placenta	SA: 84 IA: 16	SA: 57 IA: 31	PCR	Multivariable logistic regression analysis, comparison of SA to IA (best possible control), confirmation by sequencing	Small sample size, imbalance between cases and controls, study including African Americans, HPV-related disease history unclear, gestational age of controls being greater

[52]	Japan	Pregnant women healthy, unselected	1st, 2nd, or 3rd trimester	Cervix	1183	12.5	PCR	Large cohort	HPV-related disease history unclear, sample collection at different time points

[53]	USA	Pregnant women clinically indicating amniocentesis, intact membranes	NA	Amniotic fluid	142	0	PCR	Large cohort, transabdominal sampling (no birth canal contamination)	HPV-related disease history unclear, sampling time point not mentioned, highly selected group of women, unclear how/when they deliver

[54]	Mexico	Pregnant women healthy	1st, 2nd, or 3rd trimester	Cervix	274	37.1	HCA	Large cohort, unconditional/conditional logistic regression	Self-sampling, HPV-related disease history unclear, sample collection at different time points, HCA restricted to 13 HR-types

[55]	China	Pregnant women asymptomatic (cervical)	1st, 2nd, or 3rd trimester	Cervix, amniotic fluid, umbilical cord blood	116	Cervix: 36.2	PCR	Large cohort	Sample collection at different time points

[13]	Austria	Pregnant women healthy, uncomplicated pregnancy, undergoing cesarean section	37.1–40.2	Cervix, placenta, amniotic fluid, umbilical cord blood	153	Cervix: 36.6 Placenta: 5.2 Amniotic fluid: 0 Umbilical cord blood: 0	HCA, PCR	Large cohort, univariant/multivariant logistic regression analysis	Highly selected group of women, placenta swabs (quality of material)

[56]	USA	Pregnant women unselected	35 and 39	Cervix	577	29	PCR	Large cohort, logistic regression analysis, confirmation by sequencing, pap smear at study entry	No inclusion and exclusion criteria, women with history of HPV-related lesions included

[28]	Croatia	SA normal cervix	4–19	Placenta	108	7.4	PCR	Large cohort, only women with normal cervix	49.1% having a miscarriage before, 35.2% having abnormal karyotype, possible contamination due to curettage, positive results only with HPV16 and 18 specific primers

[57]	China	Pregnant women unselected, regardless of sexual history or cervical disease	1st, 2nd, or 3rd trimester	Cervix	308	10.1	PCR	Large cohort, confirmation by sequencing, age-matched controls	Sample collection at different time points, inclusion of women regardless of sexual history or cervical diseases

[22]	Austria	Pregnant women assigned for CVS or placental biopsy	9.6–31.3	Cervix, placenta	Cervix: 179 Placenta: 147	Cervix: 24.6 Placenta: 0	HCA, PCR	Large cohort, univariant/multivariant logistic regression analysis, transabdominal sampling (no birth canal contamination)	Highly selected group of women, unusual PCR primers (E6), unclear how/when they delivered, analysis of placenta by PCR, cervix by HCA

[58]	France, Switzerland, Germany	Pregnant women assigned for amniocentesis due to maternal or fetal abnormalities	14–25	Amniotic fluid	238	12	PCR, Southern blot	Large cohort, transabdominal sampling (no birth canal contamination), confirmation by Study strength	Highly selected group of women, HPV-related disease history unclear, samples collected in three countries, unclear how/when they deliver

[59]	Italy	Pregnant women healthy, negative pap smear at first trimester	36–39	Cervix	711	5.2	PCR	Large cohort, pap smear at study entry, no history of HPV-related lesions	Sampling method not mentioned, vaginal and cesarean deliveries

[60]	Greece	SA <20 weeks of gestation	6–20	Aborted product of conception	102	0	PCR	Large cohort	11 women have had previous SA, 3 cases with other diseases, GP5/6 primer (low sensitivity?), HPV-related disease history unclear

[61]	China	Pregnant women singleton	36–40	Cervix	301	22.6	PCR	Large cohort, confirmation by Study strength	Study including vaginal and cesarean deliveries, women with abnormal pap smear included, used specific E6 PCR primers only

[62]	France, Germany	SA: NA IA: for social indication	NA	Aborted product of conception	SA: 27 IA: 1	SA: 70.4 IA: 100	PCR	—	Small sample size, HPV-related disease history unclear, sampling time point not mentioned, no inclusion and exclusion criteria, PCR primer with low sensitivity

[63]	Italy	Pregnant women healthy, negative pap smear at entry	36–39	Cervix	752	5.4	PCR	Large cohort, control for confounders, logistic regression analysis, confirmation by Study strength	Sampling method not mentioned

[64]	Finland	Pregnant women healthy	At birth	Cervix	86	30.2	PCR	Confirmation by Study strength and sequencing	Possible sampling error, contamination, multiple HPV infection, inclusion of women with signs of cervical HPV infection, vaginal and cesarean deliveries

[18]	USA	SA and IA first trimester	1st trimester	Aborted product of conception	SA: 25 IA: 15	SA: 60 IA: 20	PCR	Comparison of SA to IA (best possible control), confirmation by dot blot hybridization	Small sample size, possible contamination from cervix and vagina, HPV-related disease history unclear

[65]	USA	Pregnant women healthy	NA	Cervix	114	34.2	Southern blot	Large cohort, logistic regression analysis	History of CIN not used as exclusion criteria, sampling time point not mentioned, study mostly including African Americans and Hispanics, Bronx → low socioeconomic group, Southern Blot (HPV11, 16, 18 only), unclear how/when they deliver

[66]	Germany	Pregnant women uncomplicated pregnancies	1st, 2nd, and 3rd trimester, postpartum	Cervix	108	13.9	HCA	Large cohort, logistic regression analysis, age-frequency matched controls	Specimens instead of patients, sample collection at different time points, commercial HPV detection kit (6 HPV-types only)

[67]	Hungary	Pregnant women cytologically and colposcopically healthy	1st, 3rd trimester, postpartum	Cervix	39	31	PCR	Cytologically and colposcopically healthy women	Small sample size, 8 deliveries being preterm, HPV-related disease history unclear, sample collection at different time points, outpatient clinic → low socioeconomic group

[68]	USA	Pregnant women healthy, in first trimester, ≥18 years of age	1st trimester	Cervix	245	31	HCA	Large cohort, univariant risk analysis/multiple logistic regression analysis, confirmation by Study strength	Study including African-Americans, HCA sensitivity limited to 14 HPV-types

ND = normal deliveries, PD = preterm deliveries, IA = induced abortion, SA = spontaneous abortion.

**Table:** (a) HPV prevalence in spontaneous abortion

	HPV positive cases/all cases (%)						Geographical origin	HPV detection method	References
	Cervix			Placenta					
	*n*	HPV positive cases	%	*n*	HPV positive cases	%			
Spontaneous abortion				84	48	57.1	USA	PCR	Srinivas et al. 2006 [29]
	139	34	24.5				Latin America	PCR	Conde-Ferráez et al. 2013 [36]
				51	9	17.7	Europe	PCR	Skoczyński et al. 2011 [43]
				108	8	6.5	Europe	PCR	Matovina et al. 2004 [28]
				102	0	0	Europe	PCR	Sifakis et al. 1998 [60]
				27	19	70.4	Europe	PCR	Malhomme et al. 1997 [62]
				25	15	60.0	USA	PCR	Hermonat et al. 1997 [18]

**Total**	**139**	**34**	**24.5**	**397**	**99**	**24.9**			

**Table:** (b) HPV prevalence in spontaneous preterm delivery

	HPV positive cases/all cases (%)						Geographical origin	HPV detection method	References
	Cervix			Placenta					
	*n*	HPV positive cases	%	*n*	HPV positive cases	%			
Spontaneous preterm delivery	45	7	15.6				Asia	Hybrid capture assay	Cho et al. 2013 [35]
				30	15	50.0	USA	PCR	Gomez et al. 2008 [20]
	70	47	67.1				USA	Hybrid capture assay	Zuo et al. 2011 [21]

**Total**	**115**	**54**	**47.0**	**30**	**15**	**50.0**			
